# Transcriptome Profiling Reveals the EanI/R Quorum Sensing Regulon in *Pantoea Ananatis* LMG 2665^T^

**DOI:** 10.3390/genes9030148

**Published:** 2018-03-07

**Authors:** Siphathele Sibanda, Stanford Kwenda, Collins K. Tanui, Divine Y. Shyntum, Teresa A. Coutinho, Lucy N. Moleleki

**Affiliations:** 1 Department of Microbiology and Plant Pathology, Forestry and Agricultural Biotechnology Institute (FABI), Faculty of Natural and Agricultural Sciences, University of Pretoria, 0002 Pretoria, South Africa; siphatheled@gmail.com (S.S.); stankwenda@gmail.com (S.K.); collinstonui@gmail.com (C.K.T.); dshyntum@gmail.com (D.Y.S.); lucy.moleleki@up.ac.za (L.N.M.); 2 Centre for Microbial Ecology and Genomics (CMEG), Faculty of Natural and Agricultural Sciences, University of Pretoria, 0002 Pretoria, South Africa

**Keywords:** *Pantoea ananatis*, quorum sensing, acyl homoserine lactones, regulon, RNA-seq, LMG 2665^T^

## Abstract

*Pantoea ananatis* LMG 2665^T^ synthesizes and utilizes acyl homoserine lactones (AHLs) for signalling. The complete set of genes regulated by the EanI/R quorum sensing (QS) system in this strain is still not fully known. In this study, RNA-sequencing (RNA-seq) was used to identify the EanI/R regulon in LMG 2665^T^. Pairwise comparisons of LMG 2665^T^ in the absence of AHLs (Optical density (OD)_600_ = 0.2) and in the presence of AHLs (OD_600_ = 0.5) were performed. Additionally, pairwise comparisons of LMG 2665^T^ and its QS mutant at OD_600_ = 0.5 were undertaken. In total, 608 genes were differentially expressed between LMG 2665^T^ at OD_600_ = 0.5 versus the same strain at OD_600_ = 0.2 and 701 genes were differentially expressed between LMG 2665^T^ versus its QS mutant at OD_600_ = 0.5. A total of 196 genes were commonly differentially expressed between the two approaches. These constituted approximately 4.5% of the whole transcriptome under the experimental conditions used in this study. The RNA-seq data was validated by reverse transcriptase quantitative polymerase chain reaction (RT-qPCR). Genes found to be regulated by EanI/R QS were those coding for redox sensing, metabolism, flagella formation, flagella dependent motility, cell adhesion, biofilm formation, regulators, transport, chemotaxis, methyl accepting proteins, membrane proteins, cell wall synthesis, stress response and a large number of hypothetical proteins. The results of this study give insight into the genes that are regulated by the EanI/R system in LMG 2665^T^. Functional characterization of the QS regulated genes in LMG 2665^T^ could assist in the formulation of control strategies for this plant pathogen.

## 1. Introduction

*Pantoea ananatis* is an ubiquitous, Gram-negative, yellow pigmented, rod shaped bacterium that has been described as an “emerging pathogen” of global importance [1]. This bacterium infects plants and is also capable of surviving in different environments [1]. This pathogen causes disease symptoms in a number of economically important plants such as *Eucalyptus* [1,2], staple food crops, namely maize, rice and sorghum [3] as well as cash crops, for example, honeydew melons, pineapples and onions [1]. Quorum sensing (QS) has been described as a pathogenicity determinant of *P. ananatis* [4,5], together with Type 6 Secretion System (T6SS) [6,7] and motility [8]. Unlike many other plant pathogens, *P. ananatis* does not possess Type 2 Secretion System (T2SS) and Type 3 Secretion System (T3SS) [9]. Thus, it is likely that other yet to be identified factors are involved in the pathogenicity of this bacterium.

Quorum sensing, a density dependent communication mechanism between bacterial cells in a population, is achieved by sensing the presence of accumulated QS signals called autoinducers [10]. In many Gram-negative bacteria, the acylated homoserine lactones (AHLs) are used as communication signals and expression of LuxI protein is required for the production of AHLs [11]. The LuxI/R QS regulon has been identified in a number of phytopathogens including *P. stewartii* subspecies (subsp.) *stewartii* [12], a close relative of *P. ananatis*. The draft genome sequence of *P. ananatis* LMG 2665^T^ was reported recently and contains 4893 genes, including 4787 protein coding regions [13].

Previously, we generated a QS mutant 2665^T^ *eanΔI/R* and showed that the EanI/R QS system is important for pathogenicity and biofilm formation [5]. To date, no transcriptome-wide studies have been conducted to investigate the QS regulons in any strain of *P. ananatis*. In the current study, we used transcriptome profiling through RNA-sequencing (RNA-seq) to identify the full set of genes under EanI/R QS regulation of the type strain of *P. ananatis*. This was done by comparing gene expression between optical density (OD)_600_ = 0.2 (before QS) and OD_600_ = 0.5 (during QS) in the wild-type, *P. ananatis* LMG 2665^T^. Additionally, pairwise comparison of the transcriptional changes in the wild-type strain and its QS mutant 2665^T^*eanΔI/R* during QS at OD_600_ = 0.5 was conducted. The genes that were differentially expressed between the wild-type and EanI/R mutant at OD_600_ = 0.5 and between the wild-type at the two points (OD_600_ = 0.2 (before QS) and OD_600_ = 0.5 (during QS)) were considered to be under the influence of QS. The results showed that the EanI/R QS system regulates, under the tested conditions, a variety of processes including redox sensing, metabolism, flagella formation and flagella dependent motility, cell adhesion and biofilm formation, regulators, transport, chemotaxis and methyl accepting proteins, membrane proteins, cell wall synthesis, stress response and a large number of hypothetical proteins.

## 2. Materials and Methods

### 2.1. Bacterial Strains and Growth Conditions

The bacterial strains used in this study are *P. ananatis* LMG 2665^T^ (virulent natural isolate from pineapple obtained from Belgian co-ordinated collections of microorganisms (BCCM)/LMG, University of Gent, Gent, Belgium), LMG 2665^T^ *ean∆I/R* (*P. ananatis* LMG 2665^T^ ∆ (*eanI*, *eanR*) resistant to kanamycin) [5], *Chromobacterium violaceum* 026 (mini-*Tn*5 mutant of *C. violaceum* [14]. Bacteria were routinely cultured in M9 minimal media [15] (supplemented with 2% casein hydrolysate, 1 mM MgCl_2_) at 30 °C with a shaker (Gallenkamp, Moriguchi, Japan) at 150 rev/min.

### 2.2. Identification of RNA Sampling Points

In a previous study, we generated a QS mutant and showed that EanI/R QS is responsible for pathogenicity and biofilm formation phenotypes [5]. The growth curves of LMG 2665^T^ and LMG 2665^T^ *ean∆I/R* under the studied conditions are shown in Figure S1. Here we wanted to identify the full set of genes that are under QS control using in vitro growth conditions. We first established the time at which AHLs are produced. To do this, we grew the *P. ananatis* wild-type strain in minimal media and determined the sampling points based on AHL production using the *C.violaceum* CV026 bio reporter strain [14]. The CV026 bioassay was conducted following a protocol that was previously described by [5]. Single colonies of LMG 2665^T^ wild-type were inoculated into (150 mL) M9 salts minimal media and incubated at 30 °C with shaking at 150 rev/min. The OD_600_ of bacterial cultures was determined every hour. In addition, CV026 bioassays were performed on samples that were collected every hour. The RNA sampling points were OD_600_ = 0.2 (QS absent (absence of detectable AHLs)) and OD_600_ = 0.5 (QS present (presence of detectable AHLs)). The sampling points were at early exponential phase (OD_600_ = 0.2) and mid exponential phase (OD_600_ = 0.5) (Figure S1).

### 2.3. Total RNA Isolation

RNA samples were harvested from in vitro grown *P. ananatis* LMG 2665^T^ and 2665^T^*eanΔI/R* and designated W2-1, W2-2, W2-3 for wild-type samples at OD_600_ = 0.2; W5-1, W5-2, W5-3 for wild-type samples in the presence of detectable AHLs at OD_600_ = 0.5 and M5-1, M5-2, M5-3 for mutant samples at OD_600_ = 0.5 (Table 1). Total RNA was isolated using the RNeasy Mini Kit (Qiagen, GmbH, Hilden, Germany) following instructions from the manufacturer. Three biological replicates of each bacterial culture were analysed (Table 1 shows those samples that were used for bioinformatics analysis; samples with poor mapped reads were excluded in bioinformatics analysis). To stabilize RNA, 10 mL of RNAlater^TM^ RNA stabilization reagent was added to 5 mL of culture in M9 salts minimal media followed by overnight incubation at 4 °C. The samples were then centrifuged for 10 min at 10,000 rev/min with Eppendorf centrifuge 5810R (Sigma-Aldrich, Hamburg, Germany) Cell pellets were re-suspended in a guanidine-thiocyanate-containing lysis buffer (Buffer RLT) (Qiagen). To remove residual genomic DNA (gDNA) contamination, the gDNA wipe out buffer (Qiagen) was used following the protocol from the manufacturer. Total RNA was tested for residual gDNA by polymerase chain reaction (PCR) and gel electrophoresis (results not shown). The quantity and quality of RNA were determined using the Nanodrop (Thermo Scientific, Sugarland, TX, USA) and 2100 Bioanalyser (Agilent Genomics, Santa Clara, CA, USA). RNA samples with RNA integrity number above eight were considered to be intact and not degraded.

### 2.4. Library Construction and Sequencing

Complementary DNA (cDNA) library construction and sequencing were conducted at the Beijing Genomics Institute (BGI-Shenzhen, Shenzhen, China; http://www.genomics.cn/en/index). In brief, ribosomal RNA (rRNA) was depleted using the RiboZero Magnetic Kit (Illumina Inc., San Diego, CA, USA) and messenger RNA (mRNA) was fragmented in fragmentation buffer. Synthesis of cDNA was done using mRNA fragments as templates. Purification and resolution of short fragments for end reparation and single nucleotide addition of adenine was done using the elution buffer followed by connection of adapters. Suitable cDNA fragments were selected following agarose gel electrophoresis and used as templates for PCR. The quality control of the constructed library was determined using the Agilent 2100 Bioanalyser followed by sequencing of the library using the Illumina HiSeq 2000 platform, generating 90 bp paired-end reads.

### 2.5. RNA Sequencing Data Analysis

Preliminary quality control was performed at BGI, including removal of adaptors and poor-quality reads. Prior to read mapping using Bowtie2 [16] against *P. ananatis* LMG 2665^T^ draft genome sequence, the quality of the data set was assessed using FastQC, Version 0.11.5 [17]. *P. ananatis* LMG 2665^T^ draft genome sequence data and annotation files were obtained from the NCBI Genome database (https://www.ncbi.nlm.nih.gov/genome/). A summary of mapping statistics was obtained using bam_stat.py implemented in the RNA sequencing quality control (RSeQC) package [18]. Mapped reads were filtered based on mapping quality and only uniquely mapped reads (mapping quality > 10) were used for further analysis. High-throughput sequencing (HTSeq)-count [19] was used to make the read counts, hierarchical clustering and principal component analysis (PCA) plot were used to assess the similarity and suitability of biological replicates prior to performing differential expression analysis. DESeq2 package [20] was used to perform differential expression analysis. In order to identify those genes influenced by EanI/R QS, pairwise comparisons of the wild-type strain LMG 2665^T^ transcripts in the absence of AHLs (OD_600_ = 0.2) and in the presence of AHLs (OD_600_ = 0.5) were made. Additionally, pairwise comparisons of *P. ananatis* LMG 2665^T^ and its QS mutant at OD_600_ = 0.5 were performed. Genes with a False Discovery Rate (FDR, *q*-value) < 0.05 were considered as significantly differentially expressed, for each comparison. InterProScan5 (v5.11-51) [21] was used to assign Gene Ontology (GO) IDs to the differentially expressed genes.

### 2.6. Complementary DNA Synthesis and Quantitative Reverse Transcription Polymerase Chain Reaction Validation of RNA-Sequencing Data

Synthesis of cDNA and quantitative reverse transcription (RT-qPCR) were conducted on randomly selected genes (Table 2). The selected genes included those encoding for methyl-accepting chemotaxis protein I (*tsr*), flagellar hook-associated protein 2 (*fliD*), carbamoyl 5 phosphate synthase (*carB*), galacturan 1,4-alpha 6 and shikimate 5-dehydrogenase (*aroE)* and one that codes for glycoside hydrolase and a hypothetical protein (*WP_028715941.1*). RNA samples from two sampling points, before QS (wild-type strain at OD_600_ = 0.2) and in the presence of QS (wild-type strain at OD_600_ = 0.5) were used. Synthesis of cDNA was done using the QuantiTect Reverse Transcription kit (Qiagen Inc., Germantown, MA, USA) as described by the manufacturer. Primers for the genes used for RT-qPCR were designed using OligoPerfect™ Designer (Thermo Fisher Scientific, Waltham, MA, USA) to amplify DNA fragments between 100 base pairs (bp) to 300 bp. The primers (Inqaba Biotech, Pretoria, South Africa) are listed in Table 2. The slope of a standard curve of cycle threshold (C_T_) values against concentration of serially diluted template cDNA determines the primer efficiency. The RT-qPCR reaction mix was made up of 5 µL of Applied Biosystems SYBR Green Master Mix, 1 µL of sample cDNA and each primer. The *ffh* gene that encodes the signal recognition particle protein [22,23] was used as an internal control to normalize the gene expression levels. The *ffh* gene was stable across all samples in the RNA-seq data. 

To further validate *ffh* gene as a good housekeeping gene for data normalization in *P. ananatis*, RT-qPCR was conducted and the gene was found to be stable in both conditions, OD_600_ = 0.2 and OD_600_ = 0.5 in all samples (results not shown). The RT-qPCR reactions were performed using the QuantiStudio 12 K Flex Real-Time PCR machine (Life Technologies, Carlsbad, CA, USA). The PCR protocol was as follows: initial denaturation 50 °C for 2 min, 95 °C for 2 min, 45 cycles of 95 °C for 15 s and 60 °C for 1 min and a melt curve stage: 95 °C for 15 s, 60 °C for 1 min and 95 °C for 15 s. Three technical replicates were used per sample. The fold change in gene expression levels between sampling points, before QS (wild-type strain at OD_600_ = 0.2) and in the presence of QS (wild-type strain at OD_600_ = 0.5) were calculated using the comparative C_T_ method [24] and data normalization was done using the *ffh* gene. The fold change in RNA-seq data were calculated using 2^(log_2_FoldChange)^.

### 2.7. Accession Number(s)

The RNA-seq data from this study have been deposited in NCBI’s Gene Expression Omnibus (GEO) and are accessible through the GEO accession number, GSE87207.

## 3. Results

### 3.1. RNA Sampling Points

**Figure 1:**
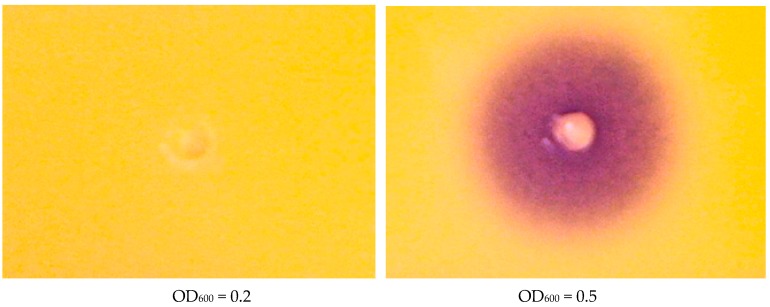
CV026 bioassay results from samples of the wild-type collected at OD_600_ = 0.2 and OD_600_ = 0.5. The purple halo indicates presence of acyl homoserine lactones (AHLs) in culture supernatant at OD_600_ = 0.5. The purple colour is a result of violacein pigment production by the CV026 bio reporter strain. The absence of purple colour at OD_600_ = 0.2 shows absence of AHLs and absence of quorum sensing.

The sampling points were determined by detection of AHLs using the CV026 assay as described in Materials and Methods. At OD_600_ = 0.2 there were no detectable AHLs from the wild-type strain indicative of absence of QS. At OD_600_ = 0.5, a purple halo by the CV026 strain indicates presence of AHLs and thus presence of QS in LMG 2665^T^ wild-type strain (Figure 1). 

### 3.2. Illumina Sequencing and Read Mapping

To determine the transcriptome wide gene expression profiles of *P. ananatis* LMG 2665^T^, cDNA libraries from LMG 2665^T^ were synthesized at OD_600_ = 0.2 (in the absence of detectable AHLs) and in the presence of AHLs (OD_600_ = 0.5). For 2665^T^*eanΔI/R,* the cDNA library was synthesized at OD_600_ = 0.5. The cDNA libraries were subjected to sequencing. Three independent biological replicates per strain were sequenced. Approximately, 20 million raw paired-end reads were produced per sample (Table 1). About 90% of these reads were successfully mapped to the *P. ananatis* LMG 2665^T^ genome sequence (Table 1).

### 3.3. Quantitative Reverse Transcription Polymerase Chain Reaction Validation of RNA-Sequencing Data

**Figure 2:**
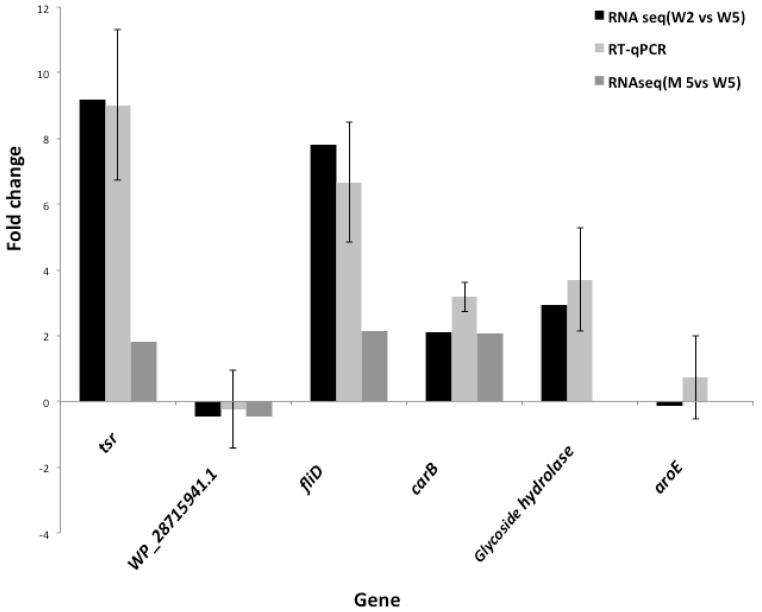
RT-qPCR validation of RNA-sequencing (RNA-seq) data using six selected genes and wild-type, *P. ananatis* LMG 2665^T^ RNA samples. The fold change in gene expression levels between sampling points, before Quorum Sensing (QS) (wild-type strain at OD_600_ = 0.2) and in the presence of QS (wild-type strain at OD_600_ = 0.5) were calculated using the comparative cycle threshold (C_T_) method [24] and data normalization was done using the *ffh* gene. The RT-qPCR results indicate fold change as cells shifted from before quorum sensing (OD_600_ = 0.2) to during quorum sensing (OD_600_ = 0.5) in LMG 2665^T^. The fold change in RNA-seq data were calculated using 2^(log_2_FoldChange)^. Error bars represent the range of relative expression calculated using 2^−(ΔΔC_T_ ± StandardDeviation)^. Triplicates were used per biological sample. The genes *aroE* and the one encoding for glycoside hydrolase were not differentially expressed in RNA-seq data (M5 versus W5).

The RT-qPCR results were in agreement with RNA-seq data, thus confirmed the reliability of the sequencing data (Figure 2). The following *aroE* and *WP_028715941.1* were not differentially expressed genes (DEG) (Figure 2), they showed no change in expression under the tested conditions. These genes were simply included as control genes.

### 3.4. Identification of the EanI/R Quorum Sensing Regulon of Pantoea Ananatis LMG 2665^T^

**Figure 3:**
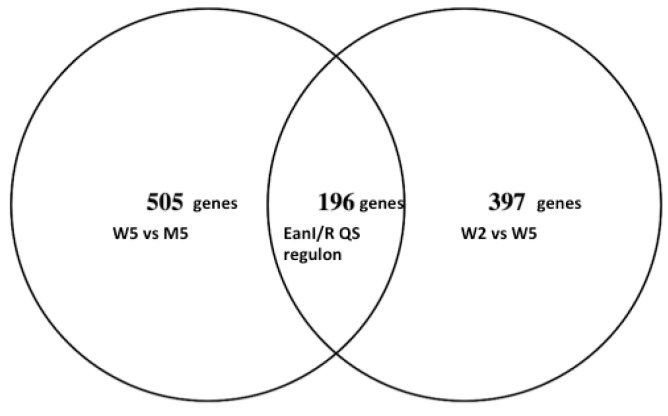
Identification of the EanI/R QS regulon**.** The genes that were differentially expressed between the sampling points before QS and during QS (W2 versus W5) that were also differentially expressed between the wild type and its QS mutants (M5 versus W5) were considered to be under QS regulation. A total of 196 genes were found to be influenced by EanI/R QS system. The number of genes in W2 versus W5 in the Venn diagram is 593 since 15 genes in W2 versus W5 data set encode for proteins with unknown protein ID. The 15 genes have protein IDs indicated as not available (N/A) in Table S2.

To unravel the QS regulon in *P. ananatis* LMG 2665^T^, two approaches were used. Firstly, genes that were differentially expressed in the wild-type LMG 2665^T^ strain between OD_600_ = 0.2 (W2) and OD_600_ = 0.5 (W5) were determined. A total of 608 genes were differentially expressed between the wild-type strain before QS and after the onset of QS (Table S1; W2 versus W5). In the second approach, pairwise comparisons of transcriptional changes between the wild-type LMG 2665^T^ (at OD_600_ = 0.5 indicating presence of QS) and the mutant 2665^T^ *eanΔI/R* (at OD_600_ = 0.5, without QS) were conducted. A total of 701 genes were differentially expressed between the two strains at OD_600_ = 0.5 (Table S2; M5 versus W5). We hypothesized that the genes that are differentially expressed in the wild-type strain between the two points (before QS and during QS) as well as between the two strains (wild-type and mutant at OD = 0.5) are influenced by EanI/R QS (Figure 3). Using this approach, we identified a total of 194 genes (excluding two genes encoding for ribosomal proteins) that were commonly differentially expressed in both approaches (Figure 3). This set of genes was selected for further analysis and is here referred to as the EanI/R QS regulon. In this regard, genes found to be regulated by EanI/R QS constituted approximately 4.5% of the whole transcriptome. A rather large difference in the genes identified in the W2/W5 versus W5/M5 comparison was observed, we speculate that this could be due differential expression of those genes that are important for the mutant’s survival and ability to cope irrespective of its lack QS.

### 3.5. Functional Annotation of the EanI/R Regulon in Pantoea ananatis

**Figure 4:**
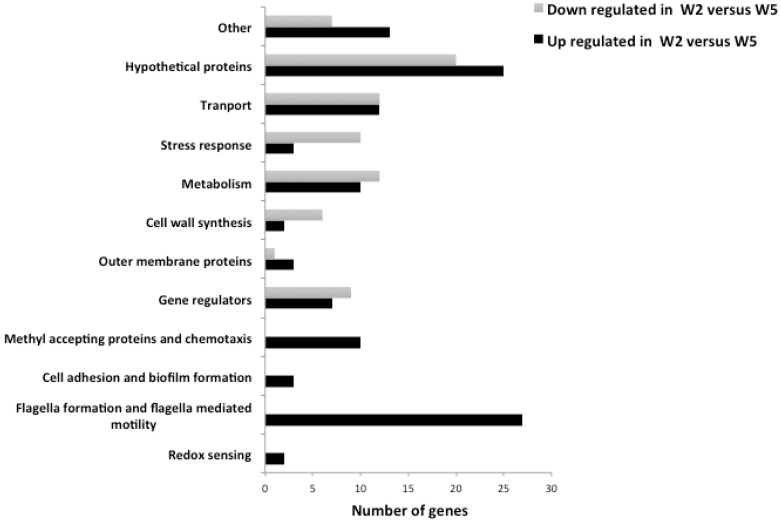
Functional categories of genes in the EanI/R QS regulon. The figure represents those genes in the EanI/R regulon that were differentially expressed in the wild type strain, LMG 2665^T^ between the two sampling points before QS and during QS. The 194 genes were grouped into different functional categories. The up regulated groups included those for flagella formation and flagella mediated motility, methyl accepting proteins and chemotaxis, redox sensing and cell adhesion. The groups with genes that were either up or down regulated included hypothetical proteins, transport, stress response, metabolism, cell wall synthesis, outer membrane proteins and regulators.

To understand the biological functions of the EanI/R QS regulon, the identified 194 DEGs were classified into functional categories. The DEGs within the EanI/R QS system were found to be those involved in redox sensing, metabolism, flagella formation and flagella dependent motility, cell adhesion and biofilm formation, regulators, transport, chemotaxis and methyl accepting proteins. In addition, other genes in the EanI/R QS regulon included those genes encoding for membrane proteins, cell wall synthesis, stress response and a large number of hypothetical proteins under the tested conditions. The list of genes in the EanI/R QS regulon and their respective log_2_ ratios in W2 versus W5 and in M5 versus W5 are shown in Table 3. The numbers of genes in each functional category are indicated in Figure 4 (the figure shows those genes differentially expressed in the wild-type strain *P. ananatis* LMG 2665^T^ between OD_600_ = 0.5 (during QS) and OD_600_ = 0.2 (before QS); W2 versus W5).

## 4. Discussion

The ability of LuxR proteins to fold, bind DNA and effect QS activity is dependent on the presence of AHLs [25]. The present study analysed the complete regulatory networks associated with the EanI/R QS system of *P. ananatis* in the presence of AHLs based on the CV026 bio reporter. The LuxI/R QS regulon of other plant pathogenic bacteria has been elucidated, for example, in *P. stewartii* subsp. *stewartii*, the LuxI/R regulon constituted about 8% of the transcriptome [12] whereas in *Pectobacterium atrosepticum* it constituted about 26% [26] representing the largest QS regulon in a plant pathogenic bacterium. In this study, transcriptome profiling showed that the EanI/R regulon in LMG 2665^T^ constituted about 4.5% of the entire transcriptome. The differences in the percentage coverages of QS regulons in different bacteria could be due to differences in bacterial species and experimental conditions. For example, *P. ananatis* has a limited number of pathogenicity factors since it neither possesses a T3SS and T2SS nor does its genome encode genes of known phytotoxins [9,27]. The success of host infection and disease development lies on the effective regulation of pathogenicity factors throughout the infection process. Importantly, it has been shown that QS regulates pathogenicity in *P. ananatis* strain LMG 2665^T^ [5] and in *P. ananatis* SK-1 [4], indicating that AHLs play a pivotal role in disease caused by *P. ananatis*.

Motility is a critical pathogenicity determinant in plant pathogenic bacteria including *Dickeya dadantii* [28], *Ralstonia solanacearum* [29] and *Pectobacterium carotovorum* subsp. *carotovorum* [30]. Moreover, a recent study showed that in *P. ananatis* motility contributes to pathogenesis and biofilm formation [8]. However, up to now there has been no information regarding QS regulation of motility in this bacterium. This study showed that the EanI/R QS positively regulates genes that encode for flagella formation and flagella mediated motility, methyl accepting proteins and chemotaxis (Table 3 and Figure 4) in LMG 2665^T^. Notably, most genes important for flagella formation and flagella mediated motility as well as methyl accepting and chemotaxis proteins were up regulated, they showed log_2_ ratios that are close to or above 2 in LMG 2665^T^ as it shifted from before QS to presence of QS (Table 3). It is thus possible that, in *P. ananatis*, the genes for motility are essential for movement towards sites that are favourable for host infection and disease outbreak. Quorum sensing was found to positively regulate genes associated with flagella biosynthesis in other plant pathogenic bacteria such as *Burkholderia glumae* [31].

The ability of a bacterium to adapt to changing environment and take advantage of available nutrients is important for pathogen survival and success of infection of plant hosts [32]. Metabolism genes up regulated by EanI/R include those encoding for pyruvate oxidase (PoxB), malate: quinone oxidoreductase (Mqo) and transketolase/WP_014593932.1. The transketolase/WP_014593932.1 is an enzyme in glycolysis and pentose phosphate pathways. Pyruvate oxidase and malate:quinone oxidoreductase (Mqo) are key enzymes in pyruvate metabolism where the former is involved in controlling production of acetic acid from pyruvate. Notably, acidic environments do not favour growth and proliferation of plant pathogenic bacteria [33,34]. In some bacteria, for example, in *P. syringae* pv. *tomato* strain DC3000, Mqo was found to be required for pathogenicity and effective utilization of nutrients available in hosts [35]. It can be speculated that this enzyme could be important for pathogenicity and nutrient acquisition in LMG 2665^T^.

Amino acids serve as a carbon source in plant pathogenic bacteria [32]. The EanI/R up regulates genes encoding lysine 6-monooxygenase and ketol-acid reductoisomerase, an enzyme important for biosynthesis of valine, leucine and isoleucine, suggesting a possible utilization of these amino acids by LMG 2665^T^. Furthermore, the present study shows that transport of various metabolic products is regulated by EanI/R QS systems (Table 3). Transport systems in plant pathogenic bacteria are essential for acquisition of nutrients in nutrient poor niches [32]. The regulation of genes for metabolism and transport by EanI/R suggests that this system could be important for survival of the pathogen in nutrient poor niches. In addition, the present study shows that the gene *wza* important for polysaccharide biosynthesis is influenced by EanI/R QS in LMG 2665^T^. Importantly, in *P. ananatis,* AHLs were found to regulate exopolysaccharides production [36].

Genes associated with iron acquisition and siderophore biosynthesis including *alcaA, lucA* and siderophore-interacting protein/WP_014606889.1 are up regulated by EanI/R QS in LMG 2665^T^ (Table 3). In *P. *stewartii** subsp. *stewartii*, mutation of genes for siderophore biosynthesis, transport and iron acquisition resulted in impaired motility and pathogenicity [37]. In addition, iron has been shown to be important for pathogenicity in bacteria such as *Pectobacterium* spp. [38] and *Erwinia* spp. [39]. The ability to outcompete other micro-organism in iron acquisition is important for survival. Given the importance of genes encoding for iron acquisition and siderophore biosynthesis in other plant pathogenic bacteria, the role of those genes in LMG 2665^T^ that are important for iron uptake and siderophore biosynthesis that were found to be QS regulated in this study deserves future investigation.

Bacterial attachment is the initial step in biofilm formation and cell adhesion in several bacterial species. It has been shown that AHL QS regulates biofilm formation [4,5]. The present study shows that the EanI/R QS system up regulates genes important for cell adhesion and biofilm formation (Table 3 and Figure 4). The *fliD* gene is important for mucus specific cell adhesion and is up regulated by EanI/R. The interaction of flagella with mucus is well documented in non-plant pathogenic bacteria, however not much research has been done on the interaction of mucilage (an analogous of mucus in plants) [40] with flagella in plant pathogens. The compelling up regulation of *fliD* gene and flagella genes observed in this study should be an incentive for investigation of a possible interaction of these proteins that are encoded by these genes in plant pathogens.

Quorum sensing regulates stress response genes in other plant pathogenic bacteria including *P. stewartii subsp. stewartii* [12], notably the universal stress proteins (UspA and UspB). The EanI/R QS system positively and negatively influences response to stress. Stress response genes that are upregulated by EanI/R include the gene encoding for carbamoyl phosphate synthase large subunit that is important for detoxification of ammonia and *hslV* important for response to heat shock (Table 3). Stress related genes that are down regulated by EanI/R include flavin adenine dinucleotide/nicotinamide adenine dinucleotide phosphate FAD/NAD(P) binding domain-containing protein important for response to oxidative stress, exodeoxyribonuclease I, class C beta-lactamase, GNAT family N-acetyltransferase NUDIX hydrolase (Table 3). The EanI/R QS system up regulates 3-oxoacyl-ACP reductase and oxidoreductase (Table 3), the two genes are involved in redox sensing that is important in sensing environmental changes [41], an important attribute for survival and adaptation.

Transcriptome profiling showed that the EanI/R up regulates expression of other gene regulators such as Fis family transcriptional regulator, transcriptional regulator/WP_028714798.1, transcriptional regulator/WP_013027687.1, *proQ* and *luxI* (Table 3). Most of the gene regulators that are influenced by EanI/R QS are those important for virulence, nutrients utilisation and response to changes in the environment. The EanI/R QS down regulates some regulators including the LysR type and transcriptional repressor (*IclR*) under the tested conditions. LysR transcriptional regulators are known for regulating pathogenicity related genes in other bacterial plant pathogens such as *D. dadantii* [42] whereas LcIR regulates pathogenicity in *P. carotovorum* subsp. *carotovorum* [43]. It is possible that EanI/R regulates some pathogenicity genes indirectly by regulating other transcriptional regulators in *P. ananatis* LMG 2665^T^. The role of these regulators in pathogenicity of LMG 2665^T^ merits investigations.

Membrane proteins are upregulated by the EanI/R system, for example *lpoA* and the ligand gated channel protein that helps facilitate entry of iron into cells whilst *ompC* is down regulated (Table 3). OmpC is a non-selective membrane porin that allows entry of all substances, including antibiotics and other toxic substances, into cells [44]. It can be reasoned that EanI/R QS down regulates *ompC* in order to protect the bacterial cells from toxic substances. The EanI/R QS system up regulates genes important for cell wall synthesis and peptidoglycan, an important component of cell walls. These include endopeptidase and *D-*alanyl-*D-*alanine carboxypeptidase (Table 3). Furthermore, genes coding for penicillin-binding protein 2, FtsI, MurJ, *D-*alanyl-*D-*alanine carboxypeptidase, N-acetylmuramoyl-*L-*alanine amidase and murein transglycosylase B are down regulated in W2 vs. W5. The regulation of outer membrane proteins by QS was noted in other plant pathogenic bacteria including *Xanthomonas citri* subsp. *citri* [45]. It can be speculated that, in LMG 2665^T^, the EanI/R QS modulates the outer membrane proteins and genes associated with cell wall synthesis in order to prevent the entrance of antimicrobial compounds that could be fatal to the pathogen. A large number of hypothetical proteins, were up regulated by the EanI/R system. Furthermore, a substantial number of hypothetical proteins, were down regulated by the EanI/R system (Table 3). The hypothetical proteins that are influenced by QS form the basis for future work, where an understanding of their biological role could lead to better knowledge of the role of QS gene regulation in *P. ananatis*.

A recent transcriptome analysis study showed that QS regulates conjugative transfer in *Agrobacterium tumefaciens* [46]. Transcriptome profiling showed that the EanI/R up regulates a conjugal transfer gene *traF*, a gene important for plasmid transfer in *P. ananatis* LMG 2665^T^ (Table 3). Some plasmids encode fitness traits required to colonize a given niche and bacteria can take up plasmids from neighbouring cells in a biofilm. Other genes upregulated by EanI/R include *srfB, maa, dusB* and those encoding for N_6_-hydroxylysine O-acetyltransferase, aminotransferase, acyl—CoA ligase, dTDP-4-dehydrorhamnose 3,5-epimerase, peptidase C39 and cytochrome ubiquinol oxidase subunit II. The cytochrome ubiquinol oxidase subunit II is important for survival in oxygen limited environments [47], possibly in a biofilm. Peptidase C39 is a bacteriocin processing enzyme, bacteriocin are antibiotics secreted by some bacteria that inhibit growth of other bacteria. This suggests the involvement of QS in regulation of a trait important for competitive advantage in LMG 2665^T^.

## 5. Conclusions

A small number of genes have been functionally characterized and found to play a role in pathogenicity of *P. ananatis* [4,5,6,8,36,48]. The present study provides information on EanI/R QS regulon in LMG 2665^T^ and adds to the list of genes that could be essential for pathogenicity and survival of this pathogen. This study provided a broader picture of the role of the EanI/R QS in *P. ananatis* LMG 2665^T^. Future studies are aimed at functional characterization of those QS regulated genes that have not yet been studied in LMG 2665^T^ as well as investigation of this QS system in planta or in the presence of a plant tissue. Such knowledge could help in the formulation of control strategies for this plant pathogen.

## Figures and Tables

**Table 1:** Statistics of reads that mapped to *Pantoea ananatis* LMG 2665^T^ genome per sample analysed.

Sample	Total Mapped Reads (%)	Uniquely Mapped Reads (%)	RNA Integrity Number (RIN)
*P. ananatis* 2665^T^*eanΔI/R* samples			
M5-1	19,318,790	16,835,822	9.5
M5-2	20,712,376	18,833,927	9.2
M5-3	20,108,558	17,421,192	9.5
*P. ananatis* 2665^T^ samples			
W2-1	19,364,330	17,023,603	9.5
W2-2	19,483,482	17,520,238	9.6
W5-1	19,953,814	15,768,738	8.8
W5-3	20,417,384	18,445,793	9.1

* Samples M5-1, M5-2 and M5-3 represent biological replicates for RNA isolated from 2665^T^*eanΔI/R* at OD_600_ = 0.5. Samples W2-1, W2-2 represent biological replicates for RNA samples isolated from 2665^T^ at OD_600_ = 0.2, samples W5-1, W5-3 are biological replicates for RNA isolated at OD_600_ = 0.5 from 2665^T^. OD: Optical density; M: Mutant; W: Wild-Type.

**Table 2:** Primers used for quantitative reverse transcription polymerase chain reaction (RT-qPCR).

Gene Name	Protein ID	Primer Name and Sequence	Source
Methyl-accepting chemotaxis protein I (*tsr*)	WP_014605659.1	TsRF CATGAATGAGATCGTCAGTGCG	This study
		TsRR GTTGTGTTACGCGATCCATCTC	
Flagellar hook-associated protein 2 (*fliD*)	WP_014593941.1	FliDF CAAATGATGGCAGTCTGTCGC	This study
		FliDR GTGATCGACACGCCGTTAATC	
Carbamoyl-phosphate synthase (*carB*)	WP_014592889.1	CarBF GATCCGAAAGTCCACCTTG	This study
		CarBR GATTGAATACGCCGTCCAC	
Hypothetical protein	WP_028715941.1	HypF CAACTGGCGGACTACCAAC	This study
		HypR GCCCTGACCAGTAATTGTCAG	
Shikimate 5-dehydrogenase (*aroE*)	WP_014606596.1	ShikiF CGACAGCGTTATTCTGACC	This study
		ShikiR AATAAGCTCAGGACGCAGG	
Glycoside hydrolase	WP_028715822.1	GlyF GCGTTGCTACCGCAAATCAAG	This study
		GlyR GTAACACCTTGCGTGTGACC	

**Table 3:** Genes regulated by the EanI/R quorum sensing system in *P. ananatis* LMG 2665^T^. Genes were identified by pairwise comparison between wild-type at OD_600_ = 0.2 (no QS) and wild type at OD_600_ = 0.5 (during QS) (W2 versus W5) and pairwise comparison of the wild-type at OD_600_ = 0.5 and QS mutant at OD_600_ = 0.5 (M5 versus W5).

Functional Category	Gene or Protein Encoded	Log_2_ Ratio	
		W2 vs. W5	M5 vs. W5
Genes up regulated between the wild-type strain LMG 2665^T^ in the presence of AHLs (at OD_600_ = 0.5) versus the same strain in the absence if AHLs (OD_600_ = 0.2)			
Redox sensing	3-oxoacyl-ACP reductase	1.833	1.638
	Oxidoreductase	2.115	1.93
Metabolism	pyruvate oxidase	1.156	1.217
	malate:quinone oxidoreductase	1.42	0.561
	transketolase/WP_014593932.1	1.401	1.588
	lysine 6-monooxygenase	1.351	0.966
	ketol-acid reductoisomerase	1.187	0.888
	*alcA*	1.532	0.869
	*IucA*	2.106	1.444
	siderophore-interacting protein (WP_014606889.1)	1.686	1.014
	pyridine nucleotide-disulphide oxidoreductase	0.845	−0.738
	*Wza*	0.971	−0.984
Flagella formation and flagella mediated Motility	*fliA*	2.837	0.848
	*flgK*	2.447	0.795
	*flagellin/*WP_014593940.1	3.053	0.952
	*fliZ*	2.607	1.012
	*fliL*	2.336	0.797
	*fligL*	2.2	0.881
	*fliM*	2.032	0.699
	*flhA*	2.086	1.488
	*flgG*	2.39	1.855
	*flagellar basal body P-ring protein*	2.104	1.542
	*fliS*	2.681	1.161
	*fliE*	2.293	1.921
	*flgN*	1.325	0.468
	*flgA*	2.279	1.357
	*flagellin/WP_013027859.1*	1.6	4.051
	*flagellar basal body rod modification protein*	2.779	1.991
	*motB*	1.728	0.595
	*flit*	2.475	1.313
	*flgF*	2.508	1.697
	*flgJ*	1.934	1.267
	*flhB*	2.199	1.559
	*flgE*	2.392	1.892
	*flgC*	2.743	2.05
	*flgL*	2.201	0.881
	*flagellar basal body L-ring protein*	2.487	1.699
	*flgB*	2.875	1.96
	*flip*	1.89	0.595
Cell adhesion and biofilm formation	*fliD*/WP_014593941.1	2.93	1.104
	*bcsO*	1.136	0.699
	*yhjQ*	0.942	0.717
Gene regulators	*luxR*	0.816	1.276
	Fis family transcriptional regulator	0.874	0.961
	transcriptional regulator/WP_028714798.1	1.669	0.877
	transcriptional regulator/WP_013027687.1	0.589	0.639
	*proQ*	2.497	0.534
	*luxI*	0.734	6.21
	*dgdR*	1.747	−0.55
Methyl accepting proteins and Chemotaxis	WP_019105711.1	2.45	1.955
	WP_028714945.1	0.994	0.683
	WP_050442519.1	1.888	0.734
	WP_014605659.1	3.141	0.869
	*exbD*	2.259	1.512
	chemotaxis protein-glutamate O-methyltransferase	2.259	0.761
	WP_014605660.1	2.716	0.882
	chemotaxis response regulator (*cheZ*)	2.194	0.717
	Regulator of chemotaxis (*cheA*)	1.888	0.743
	chemotaxis response regulator (*cheY*)	2.497	0.777
Outer membrane proteins	ligand-gated channel protein	1.061	1.621
	*lpoA*	1.102	0.431
	Outer Membrane protein/ WP_026020991.1	1.806	−0.929
Cell wall synthesis	endopeptidase	2.145	0.907
	*D-*alanyl-*D*-alanine carboxypeptidase	1.618	0.953
Stress response	*hslV*	1.741	1.539
	carbamoyl phosphate synthase large subunit	1.053	1.514
	universal stress protein B	1.457	−0.751
Transport	magnesium-translocating P-type ATPase	2.884	1.793
	MFS transporter/WP_028715176.1	2.149	1.176
	pyridine nucleotide-disulphide oxidoreductase	2.32	1.387
	anion permease	2.394	1.498
	ligand-gated channel protein/WP_014595119.1	1.491	0.954
	MFS transporter/WP_028715950.1	1.363	0.958
	NCS2 family permease	0.687	0.836
	pyridine nucleotide-disulphide oxidoreductase	2.32	1.387
	glucose dehydrogenase	0.732	0.544
	WP_014593931.1/Transketolase	1.267	1.129
	sugar ABC transporter substrate-binding protein	1.686	0.847
	ferrous iron transporter A	0.928	−2.909
Hypothetical proteins	WP_013026151.1	2.51	2.214
	WP_019106310.1	1.013	0.601
	WP_014593929.1	1.724	1.689
	WP_014333251.1	1.197	0.612
	WP_014604614.1	1.65	1.154
	WP_028715174.1	1.039	0.632
	WP_013027988.1	0.902	0.654
	WP_013026097.1	1.84	1.293
	WP_013024239.1	0.998	0.882
	WP_014593930.1	0.902	0.847
	WP_028715108.1	0.703	0.814
	WP_050442523.1	0.955	1.876
	WP_013025497.1	0.671	0.711
	WP_014332946.1(putative motility protein)	3.038	0.542
	WP_013025421.1	3.227	0.923
	WP_033765947.1	2.576	1.051
	WP_028715248.1	1.979	1.442
	WP_028715109.1	1.95	1.442
	WP_014593989.1	1.376	0.594
	WP_013024991.1	1.996	−0.63
	WP_014604946.1	0.597	−0.777
	WP_028715989.1	0.77	−0.76
	WP_050442541.1	1.443	−2.11
	WP_013025383.1	1.579	−0.98
	WP_028716027.1	1.033	−0.715
Other	*srfB (virulence factor)*	1.681	1.21
	*Maa*	2.816	2.347
	N_6_-hydroxylysine O-acetyltransferase	1.74	0.936
	Aminotransferase	1.281	1.153
	acyl—CoA ligase	1.049	1.111
	dTDP-4-dehydrorhamnose 3,5-epimerase	1.467	1.13
	*traF*	1.88	0.524
	peptidase C39	0.831	1.103
	*dusB*	0.983	0.713
	cytochrome ubiquinol oxidase subunit II	0.698	1.17
	2,5-didehydrogluconate reductase A	1.438	−0.598
	aspartyl β-hydroxylase	1.016	−1.295
	DNA polymerase III subunit ε	1.574	−0.854
Genes down regulated between the wild-type strain LMG 2665^T^ in the presence of AHLs (at OD_600_ = 0.5) versus the same strain in the absence of AHLs (OD_600_ = 0.2).			
Metabolism	erythrose-4-phosphate dehydrogenase	−0.977	−0.762
	*apbE*	−0.62	−1.113
	nicotinate-nucleotide diphosphorylase	−1.559	−0.78
	dGTPase	−0.498	−0.44
	*suhB*	−0.649	−0.472
	*L-*aspartate oxidase	−1.706	−0.601
	Protease	−1.64	−0.711
	cystathionine β-lyase	−0.789	−0.575
	peptidylprolyl isomerase	−0.722	0.624
	betaine-aldehyde dehydrogenase	−2.498	0.571
	choline dehydrogenase	−2.154	0.674
	5-oxopent-3-ene-1,2,5-tricarboxylate decarboxylase	−0.837	2.096
Outer membrane proteins	*ompC/*WP_013026835.1	−1.236	−2.393
Stress response	FAD/NAD(P) binding domain-containing protein	−0.93	−1.039
	GNAT family N-acetyltransferase	−0.751	−0.596
	class C β-lactamase	−0.953	−0.743
	exodeoxyribonuclease I	−0.533	−0.487
	NUDIX hydrolase	−0.751	−0.759
	*htpG*	−1.09	0.877
	*groEL*	−0.921	0.814
	*dnaJ*	−0.84	0.742
	sulfurtransferase	−0.733	0.971
	*hslU*	−0.619	0.473
Transport	MFS transporter/WP_033765526.1	−2.103	−0.716
	sulfate transporter subunit	−1.613	−0.567
	C4-dicarboxylate ABC transporter	−1.445	−0.993
	MATE family efflux transporter	−1.266	−0.806
	*tcyP*	−0.908	−1.025
	MFS transporter/WP_028714804.1	−0.957	−0.716
	ABC transporter permease	−0.712	−0.522
	flavocytochrome *c*	−0.712	−0.934
	microcin B17 transporter	−0.954	1.872
	MFS transporter/WP_014598266.1	−1.048	1.327
	MFS transporter/WP_013024876.1	−0.701	1.31
	nickel transporter	−0.58	1.433
Gene regulators	DNA-binding response regulator	−0.6	−0.603
	*lysR*/WP_028714815.1	−1.675	−0.718
	*draR*	−0.974	−1.295
	*lclR*	−0.862	−1.004
	*pbsX*	−0.863	−0.758
	DNA-binding response regulator	−0.734	1.974
	*yqeI*	−0.747	1.789
	*cdaR*	−1.262	0.891
	*ompR*	−0.734	1.974
Hypothetical proteins	WP_028715941.1	−1.129	−1.493
	WP_014593863.1	−1.269	−0.683
	WP_028715707.1	−1.481	−1.226
	WP_026021031.1	−1.69	−1.957
	WP_028715464.1	−0.864	−1.032
	WP_014594750.1	−0.711	−0.535
	WP_028714922.1	−1.067	−1.138
	WP_014605434.1	−1.964	−1.308
	WP_014606741.1	−1.173	−0.939
	WP_028715967.1	−0.788	−0.779
	WP_028715704.1	−0.939	−0.922
	WP_013024364.1	−0.83	−0.595
	WP_028715521.1	−0.742	−0.578
	WP_028715342.1	−0.874	−1.013
	WP_050442548.1	−1.663	−1.265
	WP_026021018.1	−1.426	−0.732
	WP_014593149.1	−1.931	−1.472
	WP_013026749.1	−0.722	1.177
	WP_022622675.1	−0.63	1.053
	WP_013024276.1	−1.146	0.744
Cell wall synthesis	penicillin-binding protein 2	−0.598	1.418
	*ftsI*	−0.726	1.406
	*murJ*	−0.562	0.549
	*D-*alanyl-*D-*alanine carboxypeptidase/WP_013026332.1	−1.335	3.127
	N-acetylmuramoyl-*L-*alanine amidase	−1.08	1.914
	murein transglycosylase B	−1.155	0.608
Other	FUSC family protein	−0.918	−0.704
	quinolinate synthetase	−1.122	−0.863
	dGTPase	−0.498	−0.44
	*diaA*	−0.658	0.527
	U32 family peptidase	−0.641	0.884
	peptidylprolyl isomerase	−0.722	0.624
	K^+^/H^+^ antiporter	−0.727	0.879

## Supplementary Materials

The following are available online at http://www.mdpi.com/2073-4425/9/3/148/s1. Table S1: Genes that were DE between the wild type strain *P. ananatis* LMG 2665^T^ before QS and the same strain after the onset of QS (W2 versus W5); Table S2: Genes that were DE between the two strains LMG 2665^T^ *ean∆I/R* and *P. ananatis* LMG 2665^T^ at OD_600_ = 0.5 (M5 versus W5); Figure S1: Growth of *P. ananatis* LMG 2665^T^ and its QS mutant LMG 2665^T^ *ean∆I/R* in vitro.

## References

[1] Coutinho T.A., Venter S.N. (2009). *Pantoea ananatis*: An unconventional plant pathogen. Mol. Plant Pathol..

[2] Coutinho T., Preisig O., Mergaert J., Cnockaert M., Riedel K.-H., Swings J., Wingfield M. (2002). Bacterial blight and dieback of *Eucalyptus* species, hybrids, and clones in South Africa. Plant Dis..

[3] Cota L., Costa R., Silva D., Parreira D., Lana U., Casela C. (2010). First report of pathogenicity of *Pantoea ananatis* in sorghum (*Sorghum bicolor*) in Brazil. Australas. Plant Dis. Notes.

[4] Morohoshi T., Nakamura Y., Yamazaki G., Ishida A., Kato N., Ikeda T. (2007). The plant pathogen *Pantoea ananatis* produces *N*-acylhomoserine lactone and causes center rot disease of onion by quorum sensing. J. Bacteriol..

[5] Sibanda S., Theron J., Shyntum D.Y., Moleleki L.N., Coutinho T.A. (2016). Characterization of two LuxI/R homologs in *Pantoea ananatis* LMG 2665^T^. Can. J. Microbiol..

[6] Shyntum D.Y., Theron J., Venter S.N., Moleleki L.N., Toth I.K., Coutinho T.A. (2015). *Pantoea ananatis* utilizes a type VI secretion system for pathogenesis and bacterial competition. Mol. Plant Microbe Interact..

[7] Shyntum D.Y., Venter S.N., Moleleki L.N., Toth I., Coutinho T.A. (2014). Comparative genomics of type VI secretion systems in strains of *Pantoea ananatis* from different environments. BMC Genom..

[8] Weller-Stuart T., Toth I., de Maayer P., Coutinho T. (2017). Swimming and twitching motility are essential for attachment and virulence of *Pantoea ananatis* in onion seedlings. Mol. Plant Pathol..

[9] De Maayer P., Chan W.Y., Venter S.N., Toth I.K., Birch P.R., Joubert F., Coutinho T.A. (2010). Genome sequence of *Pantoea ananatis* LMG20103, the causative agent of *Eucalyptus* blight and dieback. J. Bacteriol..

[10] De Kievit T.R., Iglewski B.H. (2000). Bacterial quorum sensing in pathogenic relationships. Infect. Immun..

[11] Fuqua C., Parsek M.R., Greenberg E.P. (2001). Regulation of gene expression by cell-to-cell communication: Acyl-homoserine lactone quorum sensing. Annu. Rev. Genet..

[12] Ramachandran R., Burke A.K., Cormier G., Jensen R.V., Stevens A.M. (2014). Transcriptome-based analysis of the *Pantoea stewartii* quorum-sensing regulon and identification of EsaR direct targets. Appl. Environ. Microbiol..

[13] Adam Z., Tambong J.T., Lewis C.T., Lévesque C.A., Chen W., Bromfield E.S., Khan I.U., Xu R. (2014). Draft genome sequence of *Pantoea ananatis* strain LMG 2665^T^, a bacterial pathogen of pineapple fruitlets. Genome Announc..

[14] McClean K.H., Winson M.K., Fish L., Taylor A., Chhabra S.R., Camara M., Daykin M., Lamb J.H., Swift S., Bycroft B.W. (1997). Quorum sensing and *Chromobacterium violaceum*: Exploitation of violacein production and inhibition for the detection of *N*-acylhomoserine lactones. Microbiology.

[15] Minogue T.D., Trebra M.W.V., Bernhard F., Bodman S.B.V. (2002). The autoregulatory role of EsaR, a quorum-sensing regulator in *Pantoea stewartii* ssp. *stewartii*: Evidence for a repressor function. Mol. Microbiol..

[16] Langmead B., Salzberg S.L. (2012). Fast gapped-read alignment with Bowtie 2. Nat. Methods.

[17] (2017). FastQC.

[18] Wang L., Wang S., Li W. (2012). RSeQC: Quality control of RNA-seq experiments. Bioinformatics.

[19] Anders S., Pyl P.T., Huber W. (2015). HTSeq—A Python framework to work with high-throughput sequencing data. Bioinformatics.

[20] Love M.I., Huber W., Anders S. (2014). Moderated estimation of fold change and dispersion for RNA-seq data with DESeq2. Genome Biol..

[21] Jones P., Binns D., Chang H.-Y., Fraser M., Li W., McAnulla C., McWilliam H., Maslen J., Mitchell A., Nuka G. (2014). InterProScan 5: Genome-scale protein function classification. Bioinformatics.

[22] Takle G.W., Toth I.K., Brurberg M.B. (2007). Evaluation of reference genes for real-time RT-PCR expression studies in the plant pathogen *Pectobacterium atrosepticum*. BMC Plant Biol..

[23] Moleleki L.N., Pretorius R.G., Tanui C.K., Mosina G., Theron J. (2017). A quorum sensing-defective mutant of *Pectobacterium carotovorum* ssp. *brasiliense* 1692 is attenuated in virulence and unable to occlude xylem tissue of susceptible potato plant stems. Mol. Plant Pathol..

[24] Schmittgen T.D., Livak K.J. (2008). Analyzing real-time PCR data by the comparative C_T_ method. Nat. Protoc..

[25] Zhu J., Winans S.C. (2001). The quorum-sensing transcriptional regulator TraR requires its cognate signaling ligand for protein folding, protease resistance, and dimerization. Proc. Natl. Acad. Sci. USA.

[26] Liu H., Coulthurst S.J., Pritchard L., Hedley P.E., Ravensdale M., Humphris S., Burr T., Takle G., Brurberg M.-B., Birch P.R. (2008). Quorum sensing coordinates brute force and stealth modes of infection in the plant pathogen *Pectobacterium atrosepticum*. PLoS Pathog..

[27] De Maayer P., Chan W.Y., Rubagotti E., Venter S.N., Toth I.K., Birch P.R., Coutinho T.A. (2014). Analysis of the *Pantoea ananatis* pan-genome reveals factors underlying its ability to colonize and interact with plant, insect and vertebrate hosts. BMC Genom..

[28] Antúnez-Lamas M., Cabrera-Ordonez E., Lopez-Solanilla E., Raposo R., Trelles-Salazar O., Rodríguez-Moreno A., Rodríguez-Palenzuela P. (2009). Role of motility and chemotaxis in the pathogenesis of *Dickeya dadantii* 3937 (ex *Erwinia chrysanthemi* 3937). Microbiology.

[29] Yao J., Allen C. (2007). The plant pathogen *Ralstonia solanacearum* needs aerotaxis for normal biofilm formation and interactions with its tomato host. J. Bacteriol..

[30] Hossain M.M., Shibata S., Aizawa S.-I., Tsuyumu S. (2005). Motility is an important determinant for pathogenesis of *Erwinia carotovora* subsp. *carotovora*. Physiol. Mol. Plant Pathol..

[31] Jang M.S., Goo E., An J.H., Kim J., Hwang I. (2014). Quorum sensing controls flagellar morphogenesis in *Burkholderia glumae*. PLoS ONE.

[32] Fatima U., Senthil-Kumar M. (2015). Plant and pathogen nutrient acquisition strategies. Front. Plant Sci..

[33] Lee S.-W., Jeong K.-S., Han S.-W., Lee S.-E., Phee B.-K., Hahn T.-R., Ronald P. (2008). The *Xanthomonas oryzae* pv. *oryzae* PhoPQ two-component system is required for AvrXA21 activity, HRPG expression, and virulence. J. Bacteriol..

[34] Nakka S., Qi M., Zhao Y. (2010). The *Erwinia amylovora* PhoPQ system is involved in resistance to antimicrobial peptide and suppresses gene expression of two novel type III secretion systems. Microbiolo. Res..

[35] Mellgren E.M., Kloek A.P., Kunkel B.N. (2009). Mqo, a tricarboxylic acid cycle enzyme, is required for virulence of *Pseudomonas syringae* pv. *tomato* strain DC3000 on *Arabidopsis thaliana*. J. Bacteriol..

[36] Morohoshi T., Oseki K., Ikeda T. (2011). Exopolysaccharide production is influenced by sugars, *N*-acylhomoserine lactone, and transcriptional regulators RcsA and RcsB, but does not affect pathogenicity in the plant pathogen *Pantoea ananatis*. Biosci. Biotechnol. Biochem..

[37] Burbank L., Mohammadi M., Roper M.C. (2015). Siderophore-mediated iron acquisition influences motility and is required for full virulence of the xylem-dwelling bacterial phytopathogen *Pantoea stewartii* subsp. *stewartii*. Appl. Environ. Microbiol..

[38] Tanui C.K., Shyntum D.Y., Priem S.L., Theron J., Moleleki L.N. (2017). Influence of the ferric uptake regulator (Fur) protein on pathogenicity in *Pectobacterium carotovorum* subsp. *brasiliense*. PLoS ONE.

[39] Expert D. (1999). Withholding and exchanging iron: Interactions between *Erwinia* spp. and their plant hosts. Annu. Rev. Phytopathol..

[40] Rossez Y., Wolfson E.B., Holmes A., Gally D.L., Holden N.J. (2015). Bacterial flagella: Twist and stick, or dodge across the kingdoms. PLoS Pathog..

[41] De Orué Lucana D.O. (2012). Redox Sensing: Novel Avenues and Paradigms.

[42] Hérault E., Reverchon S., Nasser W. (2014). Role of the LysR-type transcriptional regulator PecT and DNA supercoiling in the thermoregulation of PEL genes, the major virulence factors in *Dickeya dadantii*. Environ. Microbiol..

[43] Thomson N.R., Nasser W., McGowan S., Sebaihia M., Salmond G.P. (1999). *Erwinia carotovora* has two KdgR-like proteins belonging to the IciR family of transcriptional regulators: Identification and characterization of the RexZ activator and the KdgR repressor of pathogenesis. Microbiology.

[44] Blair J.M., Webber M.A., Baylay A.J., Ogbolu D.O., Piddock L.J. (2015). Molecular mechanisms of antibiotic resistance. Nat. Rev. Microbiol..

[45] Moreira L., Soares M., Facincani A., Ferreira C., Ferreira R., Ferro M., Gozzo F., Felestrino É., Assis R., Garcia C. (2017). Proteomics-based identification of differentially abundant proteins reveals adaptation mechanisms of *Xanthomonas citri* subsp. *citri* during Citrus sinensis infection. BMC Microbiol..

[46] Mhedbi-Hajri N., Yahiaoui N., Mondy S., Hue N., Pélissier F., Faure D., Dessaux Y. (2016). Transcriptome analysis revealed that a quorum sensing system regulates the transfer of the pAt megaplasmid in *Agrobacterium tumefaciens*. BMC Genom..

[47] Dalsing B.L., Truchon A.N., Gonzalez-Orta E.T., Milling A.S., Allen C. (2015). *Ralstonia solanacearum* uses inorganic nitrogen metabolism for virulence, ATP production, and detoxification in the oxygen-limited host xylem environment. mBio.

[48] Morohoshi T., Ogata Y., Ikeda T. (2011). Cell aggregation is negatively regulated by *N*-acylhomoserine lactone-mediated quorum sensing in *Pantoea ananatis* SK-1. J. Biosci. Bioeng..

